# Effects of Dietary Apple Polyphenols Supplementation on Hepatic Fat Deposition and Antioxidant Capacity in Finishing Pigs

**DOI:** 10.3390/ani9110937

**Published:** 2019-11-08

**Authors:** Xiaojiao Xu, Xiaoling Chen, Zhiqing Huang, Daiwen Chen, Jun He, Ping Zheng, Hong Chen, Junqiu Luo, Yuheng Luo, Bing Yu, Jie Yu

**Affiliations:** 1 Key Laboratory for Animal Disease-Resistance Nutrition of China Ministry of Education, Institute of Animal Nutrition, Sichuan Agricultural University, Chengdu 611130, Sichuan, China; xuxiaojiao123@163.com (X.X.); xlchen@sicau.edu.cn (X.C.); chendwz@sicau.edu.cn (D.C.); hejun8067@163.com (J.H.); zpind05@163.com (P.Z.); ljqlcy@tom.com (J.L.); luoluo212@126.com (Y.L.); ybingtian@163.com (B.Y.); jerryyujie@163.com (J.Y.); 2 College of Food Science, Sichuan Agricultural University, Yaan 625014, Sichuan, China; chenhong945@sicau.edu.cn

**Keywords:** apple polyphenols, finishing pigs, hepatic fat deposition, antioxidant capacity, mechanisms, lipid profiles

## Abstract

**Simple Summary:**

Excessive fat deposition (5–10%) in the liver could lead to liver damage and nonalcohol fatty liver disease (NAFLD). However, there is no satisfactory safe and effective measure of preventive and therapeutic treatments so far. Thus, the prevention of excessive fat deposition through diet modification might be a better strategy to protect humans from metabolic diseases. Due to the anatomical and physiological similarities between humans and pigs, the present study took the finishing pig as an animal model to investigate the effects of apple polyphenols on hepatic fat deposition and antioxidant capacity and their mechanisms. The present study indicated that apple polyphenols might be an effective dietary supplementation for decreasing the excessive fat deposition in liver tissue, improving lipid profiles and increasing the antioxidant capacity of finishing pigs. This study provides a better preventive strategy to protect humans from excessive fat deposition in the liver.

**Abstract:**

Excessive fat deposition in the liver could lead to fatty liver and an increased risk of many metabolic diseases. Apple polyphenols (APPs), the major antioxidants in apples, possess wide-ranging beneficial biological functions. The present study aimed to investigate the effects of APPs on hepatic fat deposition and antioxidant capacity in finishing pigs, and their mechanisms. Results showed that APPs improved lipid profiles, increased antioxidant enzyme activities and reduced the fat deposition in the liver. In the liver, *SOD1*, *CAT*, *GPX1*, *GST*, NF-E2-related nuclear factor 2 (*Nrf2*), hormone sensitive lipase (*HSL*), carnitine palmitoyl transferase-1b (*CPT1b*), peroxisome proliferator-activated receptor α (*PPARα*), cholesterol 7α-hydroxylase (*CYP7A1*) and low-density lipoprotein receptor (*LDL-R*) mRNA levels were increased by APPs, while Kelch-like ECH-associated protein 1 (*Keap1*) mRNA level, C16:0 and C20:4n-6 proportions and Δ9-18 dehydrogenase activity were decreased. In conclusion, this study indicated that APPs might be an effective dietary supplementation for improving lipid profiles, increasing antioxidant capacities and decreasing fat deposition in the liver.

## 1. Introduction

Excessive fat deposition (5–10%) in the liver can lead to liver damage and nonalcohol fatty liver disease (NAFLD) [1,2]. Severe or long-term damage can even lead to irreversible liver disease (cirrhosis), that causes the liver to stop working properly, similar to the damage caused by alcohol abuse [1,2]. In addition to NAFLD, excessive fat deposition usually induces a wide range of metabolic diseases, such as hyperlipemia and cardiovascular disease (CVDs). Although considerable progress in preventive and therapeutic treatments has been made to fight these metabolic diseases, the effect of these measures was barely satisfactory [3]. Thus, prevention of excessive fat deposition through diet modification might be a better strategy to protect humans from metabolic diseases. Apple polyphenols (APPs), the major antioxidants in apples, have been reported to possess wide-ranging beneficial biological functions, such as antioxidant capacity [4,5], anti-inflammatory [6], hypoglycemic [7] and antiviral effects [8], and cardiovascular diseases prevention functions [9,10]. Therefore, APPs are recognized as an effective food additive and dietary supplementation in Japan [11].

Previous studies have reported that APPs have the function of reducing white adipose tissue deposition in normal-weight or obese rodents [12,13]. However, there is no published report on the effects of APPs on the fat deposition in the liver. Therefore, it is essential to further explore the fat deposition-lowering effect of APPs.

Generally, there is a dynamic balance existing between the generation and elimination of cellular-reactive oxygen species (ROS) [14]. When ROS level is increased or antioxidant capacity is decreased, excessive ROS deposition results in the damage of cell structure and induces cancers [15], diabetes [16] and aging [17]. In addition to regulating lipid metabolism, the liver processes a powerful antioxidant system to eliminate ROS through an antioxidative enzyme system and non-enzymatic antioxidants. Previous studies have reported that APPs could increase body antioxidant capacity in rodents [12,18]. However, its underlying mechanisms in the liver are rarely reported, so it still needs to be further explored.

Because of the anatomical and physiological similarities between humans and pigs, pigs are a more suitable animal model than rodents for the study of human nutrition and metabolism [19,20]. Moreover, the metabolic organs and features of finishing pigs and adults are similar, and their sizes are also proportionate [21,22]. Taking this into consideration, we investigated the effects of dietary APP supplementation on hepatic fat deposition and antioxidant capacity in a finishing pig model. Its mechanisms were also investigated.

## 2. Materials and Methods 

All animal procedures in this experiment were approved by the Committee on Animal Care Advisory of Sichuan Agricultural University, under permit No. YYS180526.

### 2.1. Animals and Diets

A total of thirty-six healthy castrated Duroc × Landrace × Yorkshire (DLY) pigs with an average body weight (BW) of 71.25 ± 2.40 kg were individually divided into three diet groups (basal diet, 0.04% APPs + basal diet, 0.08% APPs + basal diet). Each group was replicated six times using two pigs for each replication. The basal diet was based on maize–soybean meal and formulated to meet the nutrient requirements for 75–100 kg and 100–135 kg finishing pigs of the National Research Council (NRC 2012). The dietary composition of the basal diet is shown in Table 1. Additionally, APPs (purity of 98.3%) were produced by Xi’an Hao Yuan Biotechnology Co., Ltd. (Xi’an, China). All pigs had free access to feed and clean water throughout the experimental period, and the trial lasted for 49 d.

### 2.2. Sample Collection

After a 49-day period of feeding trial, blood samples from the external jugular vein of all pigs were collected after 12 h fasting, and rested at room temperature for 30 min. After being centrifuged at 3000× *g* for 10 min at 4 °C, the serum samples were collected and stored at −20 °C. Eighteen pigs (six per treatment, one per pen close to the average BW of the treatment) were selected and electrically stunned. Fresh liver samples were collected and stored at −80 °C until analysis for hepatic parameters, fatty acid profiles, ether extract content and gene expression.

### 2.3. Fat Deposition Analysis

About 50 g liver samples were sliced up, weighed, placed in a weighing bottle and reweighed. Weighing bottles were placed into a freeze dryer with a temperature of −50 °C for 48 h, and then reweighed. The difference in the weights of initial and dried samples was used to calculated the moisture percentage. Subsequently, dried samples were pulverized using a muller, and then used for fatty acid profiles and ether extract content analysis. The measurement of ether extract content was performed according to the methods of the Association of Analytical Chemists [23].

### 2.4. Serum Antioxidant and Biochemical Analysis

The activities of total antioxidant capacity (T-AOC), total superoxide dismutase (T-SOD), glutathione peroxidase (GSH-Px) and catalase (CAT), and the contents of malonaldehyde (MDA), triacylglycerol (TG), total cholesterol (T-CHO), high density lipoprotein cholesterol (HDL-C) and low density lipoprotein cholesterol (LDL-C) in serum were determined, using corresponding assay kits purchased from Nanjing Jiancheng Bioengineering Institute (Nanjing, Jiangsu, China), according to the manufacturer’s instructions.

### 2.5. Hepatic Antioxidant and Biochemical Analysis

Liver samples and precooled 0.9% saline were added proportionally at a ratio of 1:9 into a 1.5 mL centrifuge tube and subjected to sonication in an ice bath. After being centrifuged at 2500 rpm for 10 min at 4 °C, the supernatant was collected and used to measure the activities of T-AOC, T-SOD, CAT and GSH-Px, and the contents of MDA, TG and T-CHO in triplicate. The total protein of the supernatant was measured by Coomassie blue staining method, using an assay kit purchased from Nanjing Jiancheng Bioengineering Institute (Nanjing, Jiangsu, China).

### 2.6. Analysis of the Fatty Acid Profile

Lipid in the liver was extracted according to the method described by Folch et al. [24]. The extracted lipid was hydrolyzed in 2 mL KOH-methanol (*C* = 0.5 mol/L) with shaking for 1 min. The mixture was reacted in 95 °C water with continual shaking for 10 min. The free fatty acid mixture was esterified in 2 mL BF_3_-methanol solution (*W* = 10%) with shaking for 10 s. The mixture was reacted in 80 °C water with continual shaking for 20 min. Then, 1 mL n-hexane and 5 mL saturated NaCl solution were added into the mixture. After shaking for 1 min, the mixture was centrifuged at 3000 rpm for 15 min. 800 μL fatty acid methyl esters were collected and analyzed with a GC-2010 plus gas chromatography (Shimadzu, Japan).

### 2.7. Real-Time Quantitative PCR

Briefly, total RNA extraction, reverse transcription and real-time quantitative PCR were performed according to the method described by Zhang et al. [25], using RNAiso Plus reagent (TaKaRa, Dalian, China), primeScript RT reagent kit with gDNA eraser (TaKaRa) and SYBR^®^ Premix Ex Taq™ II reagents (TakaRa), respectively, according to manufacturer’s directions. The primers of real-time 

Quantitative PCR are listed in Table 2. Relative mRNA levels of related genes in the liver were calculated by the 2^−ΔΔCt^ method, with glyceraldehyde phosphate dehydrogenase (*GAPDH*) mRNA as a reference gene [26].

### 2.8. Statistical Analyses

Data, presented as mean ± SE, were analyzed by the one-way ANOVA and Duncan’s multiple range test, using statistical software SPSS 22.0 (SPSS Inc., Chicago, IL, USA). *P* < 0.05 and 0.05 ≤ *P* < 0.10 were used to determine statistical significance and tendency among results, respectively.

## 3. Results

### 3.1. Fat Deposition

**Figure 1:**
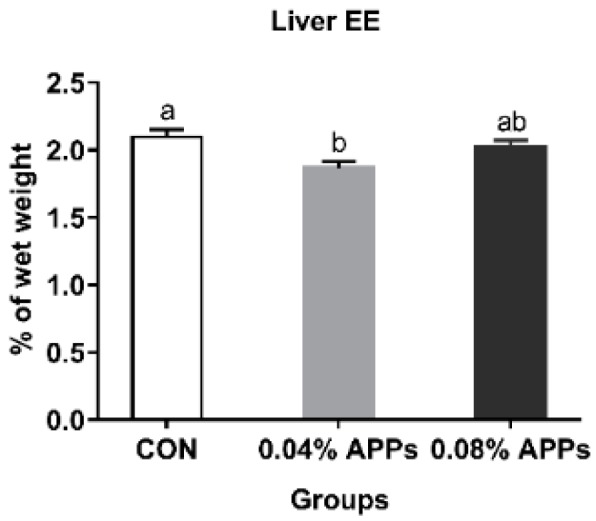
Effect of dietary APPs supplementation on liver ether extract content of finishing pigs. Results of each group are represented as the mean ± SE from six pigs. Within a panel, different letters differ significantly at *P* < 0.05. CON, basal diet; 0.04% APPs, basal diet with APPs at 0.04%; 0.08% APs, basal diet with APPs at 0.08%. APPs = Apple polyphenols; EE = Ether extract.

As shown in Figure 1, compared with the control group, the content of liver ether extract was significantly decreased (*P* < 0.05) in the 0.04% APPs diet group. There was no difference (*P* > 0.10) in fat deposition between 0.04% and 0.08% APPs diet groups (Figure 1). 

### 3.2. Serum Parameters

As shown in Table 3, dietary APPs supplementation had no effect (*P* > 0.10) on the activities of serum T-SOD and GSH-Px, as well as on the contents of LDL-C, HDL-C and MDA, but resulted in greater (*P* < 0.05) T-AOC activity and lower TG content. In addition, compared with the control group, the serum T-CHO content was remarkably reduced (*P* < 0.05) in the 0.04% APPs diet group, and the 0.08% APPs had significantly decreased (*P* < 0.05) serum CAT activity (Table 3). There was no difference (*P* > 0.10) in serum parameters between the 0.04% and 0.08% APPs diet groups (Table 3).

### 3.3. Hepatic Parameters

As shown in Table 4, dietary APPs supplementation had no effect (*P* > 0.10) on hepatic T-AOC, T-SOD and CAT activities, but resulted in lower (*P* < 0.05) MDA, TG and T-CHO contents. In addition, the hepatic GSH-Px activity was remarkably increased (*P* < 0.05) in the 0.08% APPs diet group, compared with the basal and 0.04% APPs diet groups (Table 4).

### 3.4. Hepatic Antioxidant-Related Gene mRNA Levels

As shown in Table 5, dietary APPs supplementation had significantly increased (*P* < 0.05) the hepatic *SOD1*, *CAT*, *GPX1* and *Nrf2* mRNA levels, but resulted in lower (*P* < 0.05) *Keap1* mRNA expression level. In addition, the hepatic *GST* mRNA level was significantly increased (*P* < 0.05) in the 0.08% APPs diet group, compared with the basal and 0.04% APPs diet groups (Table 5).

### 3.5. Hepatic Lipid Metabolism-Related Gene mRNA Levels

As shown in Table 6, dietary APPs supplementation had no effect (*P* > 0.10) on hepatic *ACC*, *FAS* and *HMG-CoAR* mRNA levels, but resulted in higher (*P* < 0.05) *HSL*, *CPT1b* and *CYP7A1* mRNA levels. The 0.04% APPs tended to decrease the mRNA level of *HMGCoAR* (0.05 ≤ *P* < 0.10) (Table 6). In addition, the hepatic *PPARα* and *LDL-R* mRNA levels were significantly upregulated (*P* < 0.05) in the 0.08% APPs diet group, compared with the basal and 0.04% APPs diet groups (Table 6).

### 3.6. Hepatic Fatty Acid Profiles

As shown in Table 7, pigs fed with APPs had lower (*P* < 0.05) proportions of C16:0 and C20:4n6, compared with the pigs fed with the basal diet. In addition, the Δ9-18 desaturase activity had significantly decreased (*P* < 0.05) in the 0.08% APPs diet group, compared with the basal and 0.04% APPs diet groups (Table 7).

## 4. Discussion

Excessive fat deposition in the liver is one of the manifestations of unhealthy metabolic obesity and increases the risk of NAFLD and cirrhosis [27]. Our study found that dietary APPs supplementation decreased hepatic fat deposition and amounts of TG and T-CHO in finishing pigs. Lipogenesis, lipolysis and fatty acid oxidation are the three key steps of lipid metabolism. Therefore, we explored the mechanisms of the fat-lowering action of APPs, by detecting the expression of genes related to these three steps. When lipogenesis occurs, ACC catalyzes the carboxylation of acetyl CoA, which is the rate-limiting step of de novo lipogenesis [28]. FAS controls the synthesis of palmitic acid from malonyl CoA and is the decisive factor for de novo lipogenesis in tissue [29]. When lipid mobilization occurs, HSL, as the rate-limiting enzyme for lipolysis, controls the first step in the decomposition of triglyceride, and hydrolyzes the triglyceride to diglyceride and fatty acid [30]. Subsequently, produced fatty acids enter the mitochondria and then are further oxidized to produce energy [31]. Acyl carnitine is the main form of fatty acid transported into mitochondria, and CPT-1b is a rate-limiting enzyme for fatty acid β-oxidation, and catalyzes the formation of long-chain acyl carnitine from free carnitine and acyl CoA [32,33]. PPARα can regulate the mRNA expression level of CPT-1b [31]. Therefore, PPARα and CPT1b are the marker genes for fatty acid β-oxidation. Previous study reported that APPs inhibited the Dex-induced lipogenesis in human sebocytes by decreasing *ACC* and *FAS* mRNA levels [34] and increased the mRNA levels of *PPARα* and *CPT-II* in the liver [12,35,36]. In this study, we showed that dietary APPs supplementation had no significant effect on *ACC* and *FAS* mRNA levels in liver tissue, which was inconsistent with the previous study, suggesting that the fat-lowering effect of APPs might not be mediated by hepatic lipogenesis. Moreover, we found that APPs remarkably increased *HSL*, *PPARα* and *CPT1b* mRNA levels in the liver of finishing pigs, suggesting that the fat-lowering effect of APPs might be mediated by hepatic lipolysis and fatty acid β-oxidation.

It has been reported that obesity indexes were positively correlated with the proportions of serum C16:0, C16:1n-7, C18:0, C18:3n-6, C20:3n-6, C20:4n-6 and C20:5n3, as well as the activities of Δ9 and Δ6 dehydrogenase [37]. Δ9 dehydrogenase can catalyze the dehydrogenation of C16:0 and C18:0 to C16:1 and C18:1n9, and is positively correlated with obesity [37,38]. The work of Ntambi et al. has confirmed that the decrease of Δ9 dehydrogenase activity could improve insulin sensitivity in mice [39]. The work of Zhou et al. has reported that the decrease of Δ9 dehydrogenase activity in the liver helped to inhibit hepatic fat deposition [40]. Moreover, there is a close relationship between fatty acid composition and metabolic syndromes in individuals, such as insulin resistance and diabetes [41]. The proportions of C16:0 and C20:4n-6 were reported to be associated with insulin action [41,42]. In our present study, APPs significantly decreased the proportions of C16:0 and C20:4n-6, as well as the activity of Δ9-18 dehydrogenase, suggesting that APPs could regulate fatty acid composition and increase insulin sensitivity.

As for cholesterol metabolism, HMG-CoAR, LDL-R and CYP7A1 are the marker genes in the synthesis, reabsorption and transformation of cholesterol, respectively. An amount close to 70%~80% of the cholesterol in the body is derived from endogenous biosynthesis, so limiting the synthesis of cholesterol is the most effective measure to reduce the total cholesterol [43]. HMGCoAR is the rate-limiting enzyme in the de novo synthesis of cholesterol. The work of Xu et al. has found that *HMGCoAR* mRNA level was reduced when ApoE ^−/−^ mice were orally treated with APPs [12]. In the present study, we found that dietary 0.04% APPs supplementation tended to decrease the mRNA level of *HMGCoAR*. LDL-R is located in the hepatocyte membrane and can combine with LDL to reabsorb cholesterol from circulating LDL-C. The work of Tenore et al. reported that apple polyphenolic extracts increased LDL-R binding activity in HepG2 cells [44]. Consistently, this study indicated that APPs could upregulate the mRNA level of hepatic *LDL-R* in finishing pigs. In hepatocyte, most of the cholesterol has been transformed to bile acid to maintain cholesterol homeostasis and prevent cholesterol over-accumulation in body [45]. The rate-limiting enzyme of this step is CYP7A1, which converts cholesterol to 7α-hydroxycholesterol [43]. The work of Osada et al. reported that, when rats were fed a high-cholesterol diet with 0.2%, 0.5% and 1.0% APPs, APPs could increase the activity of hepatic CYP7A1, thereby increasing the excretion of acidic steroids in feces [46]. In this study, we showed that *CYP7A1* mRNA levels in the liver increased when finishing pigs were fed a diet with APPs. Our present study indicated that APPs could increase cholesterol reabsorption and transformation, which might account for the blood cholesterol lowering effect of APPs. 

The antioxidant systems are the central defense lines to protect the body from oxidative stress and activated by multifarious bioactive substances and antioxidant related genes, including Nrf2-Keap1 pathway [47]. Nrf2 is a key activator of antioxidant enzymes related genes and suppressed by Keap1 in cells [48]. When the body suffers from oxidative stress, the Nrf2 dissociates from Keap1 and is phosphorylated, to stimulate the expression of antioxidant related enzymes, such as CAT, SOD1, GPX1 and GST [49]. Consistent with previous research in rodents [12,18], our present study indicates that apple polyphenols could significantly increase the activities of CAT, T-AOC, T-SOD, GSH-Px, and the mRNA levels of *Nrf2*, *SOD1*, *CAT*, *GPX1*, *GST*, but decrease the content of MDA. In addition, the results of our present study show that the mRNA level of *Keap1* in liver tissue was decreased by apple polyphenols, suggesting that the increased *Nrf2* expression might be partly mediated by an inhibition of *Keap1* expression.

## 5. Conclusions

Taken together, our present study indicates that APPs might be an effective dietary supplementation for improving lipid profiles and antioxidant capacities, as well as decreasing excessive fat deposition in liver tissue. As for the mechanisms of this, we provided evidence that the liver fat-lowering effect of APPs might be due to the increase in lipolysis and fatty acid oxidation, and the decrease of T-CHO by APPs might be attributed to an increase in cholesterol reabsorption and transformation. We also provided evidence that its effect on increasing antioxidant capacities might be attributed to the stimulation of the Nrf2-Keap1 system.

## Figures and Tables

**Table 1:** Composition and nutrient levels of the basal diet.

Ingredient	Content (%)	Nutrient Levels ^3^	Content
Maize	79.18	Digestible energy (Mcal/kg)	3.40
Soybean meal	16.02	Crude protein (%)	13.76
Soybean oil	1.97	Calcium (%)	0.49
Maize starch	0.15	Total P (%)	0.41
*L*-Lysine·HCl	0.34	Available P (%)	0.23
*DL*-Methionine	0.10	Digestible lysine (%)	0.78
*L*-Threonine	0.15	Digestible Met + Cys (%)	0.47
*L*-Tryptophan	0.02	Digestible Thr (%)	0.53
Limestone	0.76	Digestible Thr (%)	0.14
CaHPO_4_	0.60		
NaCl	0.30		
Choline chloride	0.10		
Vitamin premix ^1^	0.015		
Mineral premix ^2^	0.30		
Total	100.00		

^1^ Vitamin premix provided the followings per kg of basic diets: Vitamin A, 4500 IU; Vitamin D_3_, 1500 IU; Vitamin E, 12 IU; Vitamin K, 1.5 mg; Vitamin B_2_, 3.75 mg; Vitamin B_3_, 15 mg; Vitamin B_5_, 7.5 mg; Vitamin B_1_, 1.5 mg; Vitamin B_6_, 1.8 mg; Vitamin B_12_, 18mg; Folic acid, 0.75 mg; Biotin, 0.075 mg. ^2^ Mineral premix provided the followings per kg of basic diets: Fe, 40 mg; Cu, 3 mg; Mn, 2 mg; Zn, 50 mg; I, 0.14 mg; Se, 0.15 mg. ^3^ Nutrient levels were calculated values.

**Table 2:** Primer sequences used for real-time quantitative PCR.

Genes	Primer Sequence (5′–3′)	Product Size (bp)	GeneBank ID Accession No.
*GAPDH*	F: ACTCACTCTTCTACCTTTGATGCT R: TGTTGCTGTAGCCAAATTCA	100	NM_001206359
*ACC*	F: ACCGAATTGGTTCCTTTGGAC R: CCAGTCCGATTCTTGCTCCA	123	AF175308
*FAS*	F: ACACCTTCGTGCTGGCCTAC R: ATGTCGGTGAACTGCTGCAC	112	NM_001099930
*HSL*	F: CCCATCCTCTCCATCGACT R: CAGCAGTAGGCGTAGAAGCAC	83	NM_214315
*PPARα*	F: GAGTTCGCCAAGTCCATCC R: CCGTCCTTGTTCATCACAGAG	122	NM_001044526
*CPT1b*	F: TGACTCGAATGTTCCGGGAG R: AGATCTTGCAGGTCTGCTTTCA	118	NM_001007191
*HMG-CoAR*	F: GGTCAGGATGCGGCACAGAACG R: GCCCCACGGTCCCGATCTCTATG	127	NM_001122988
*CYP7A1*	F: TATAGGGCACGATGCACAGA R: ACCTGACCAGTTCCGAGATG	200	NM_001005352
*LDL-R*	F: AGAACTGGAGGCTTAAGAGCATC R: GAGGGGTAGGTGTAGCCGTCCTG	115	NM_001206354
*SOD1*	F: AGACCTGGGCAATGTGACTG R: GTGCGGCCAATGATGGAATG	102	NM_001190422
*CAT*	F: CAGATGAAGCATTGGAAGGAGC R: TTGTCTCCTATCGGATTCCCAG	83	NM_214301
*GPX1*	F: GTGAATGGCGCAAATGCTCA R: ATTGCGACACACTGGAGACC	126	NM_214201
*GST*	F: CCAACCCAGAAGACTGCTCA R: CATTCAGGTGGGCTCTTCGT	102	AB000884
*Nrf2*	F: GCCCCTGGAAGCGTTAAAC	67	XM_003133500
	R: GGACTGTATCCCCAGAAGGTTGT		
*Keap1*	F: ACGACGTGGAGACAGAAACGT	56	NM_001114671
	R: GCTTCGCCGATGCTTCA		

*GAPDH* = Glyceraldehyde phosphate dehydrogenase; *ACC* = Acetyl-CoA carboxylase; *FAS* = Fatty acid synthase; *HSL* = Hormone sensitive lipase; *PPARα* = Peroxisome proliferator-activated receptor α; *CPT1b* = Carnitine palmitoyl transferase-1b; *HMG-CoAR* = 3-hydroxy-3-methylglutaryl coenzyme A reductase, *CYP7A1* = Cholesterol 7α-hydroxylase; *LDL-R* = Low-density lipoprotein receptor; *SOD1* = Superoxide dismutase 1; *CAT* = Catalase; *GPX1* = Glutathione peroxidase 1; *GST* = Glutathione S-transferase; *Nrf2* = NF-E2-related nuclear factor 2; *Keap1 =* Kelch-like ECH-associated protein 1.

**Table 3:** Effect of dietary APPs supplementation on the serum parameters of finishing pigs.

Items	CON	0.04% APPs	0.08% APPs
**Antioxidant capacity**			
MDA, nmol/mL	0.65 ± 0.09	0.57 ± 0.07	0.48 ± 0.10
T-AOC, U/mL	0.81 ± 0.07 ^b^	1.15 ± 0.09 ^a^	1.15 ± 0.10 ^a^
T-SOD, U/mL	102.77 ± 1.19	99.36 ± 1.93	100.28 ± 1.81
GSH-PX, U/mL	416.51 ± 21.53	500.78 ± 42.09	486.06 ± 25.30
CAT, U/mL	11.09 ± 0.77 ^b^	13.15 ± 1.34 ^ab^	14.23 ± 0.77 ^a^
**Biochemistry parameters**			
T-CHO, mmol/L	3.04 ± 0.10 ^a^	2.69 ± 0.07 ^b^	2.86 ± 0.10 ^ab^
TG, mmol/L	0.46 ± 0.01 ^a^	0.36 ± 0.02 ^b^	0.36 ± 0.01 ^b^
LDL-C, mmol/L	1.34 ± 0.06	1.18 ± 0.06	1.30 ± 0.07
HDL-C, mmol/L	1.70 ± 0.06	1.72 ± 0.08	1.79 ± 0.09

^a, b^ within a row, different letters differ significantly at *P* < 0.05. APPs = Apple polyphenols; MDA = Malondialdehyde; T-AOC = Total antioxidant capacity; T-SOD = Total superoxide dismutase; GSH-Px = Glutathione peroxidase; CAT = Catalase; T-CHO= Total cholesterol; TG = Triglyceride; LDL-C = Low-density lipoprotein-cholesterol; HDL-C = High-density lipoprotein-cholesterol.

**Table 4:** Effect of dietary APPs supplementation on the hepatic parameters of finishing pigs.

Items	CON	0.04% APPs	0.08% APPs
**Antioxidant capacity**			
MDA, nmol/mg prot	2.29 ± 0.19 ^a^	1.11 ± 0.17 ^b^	1.11 ± 0.20 ^b^
T-AOC, U/mg prot	1.07 ± 0.06	1.18 ± 0.05	1.19 ± 0.08
T-SOD, U/mg prot	1.89 ± 0.08	1.78 ± 0.06	1.69 ± 0.11
GSH-PX, U/mg prot	411.58 ± 23.10 ^b^	412.41 ± 11.92 ^b^	470.95 ± 18.24 ^a^
CAT, U/mg prot	19.37 ± 0.51	18.49 ± 0.47	18.56 ± 0.27
**Biochemistry parameters**			
T-CHO, mmol/mg prot	58.55 ± 3.96 ^a^	42.48 ± 1.56 ^b^	43.98 ± 4.27 ^b^
TG, mmol/mg prot	106.34 ± 6.27 ^a^	83.61 ± 5.12 ^b^	79.86 ± 2.06 ^b^

^a, b^ within a row, different letters differ significantly at *P* < 0.05. APPs = Apple polyphenols; MDA = Malondialdehyde; T-AOC = Total antioxidant capacity; T-SOD = Total superoxide dismutase; GSH-Px = Glutathione peroxidase; CAT = Catalase; T-CHO= Total cholesterol; TG = Triglyceride.

**Table 5:** Effect of dietary APPs supplementation on hepatic antioxidant related genes mRNA levels of finishing pigs.

Items	CON	0.04% APPs	0.08% APPs
*SOD1*	1.00 ± 0.03 ^c^	1.25 ± 0.05 ^b^	1.49 ± 0.06 ^a^
*CAT*	1.00 ± 0.02 ^b^	1.37 ± 0.05 ^a^	1.28 ± 0.04 ^a^
*GPX1*	1.00 ± 0.02 ^c^	1.60 ± 0.05 ^b^	1.97 ± 0.05 ^a^
*GST*	1.00 ± 0.02 ^b^	1.21 ± 0.04 ^b^	1.65 ± 0.10 ^a^
*Keap-1*	1.00 ± 0.04 ^a^	0.80 ± 0.03 ^b^	0.77 ± 0.03 ^b^
*Nrf2*	1.00 ± 0.08 ^b^	1.76 ± 0.05 ^a^	1.86 ± 0.21 ^a^

^a, b, c^ Within a row, different letters differ significantly at *P* < 0.05. APPs = Apple polyphenols; *SOD1* = Superoxide dismutase 1; *CAT*= Catalase; *GPX1* = Glutathione peroxidase 1; *GST* = Glutathione S-transferase; *Keap1 =* Kelch-like ECH-associated protein 1; *Nrf2* = NF-E2-related nuclear factor 2.

**Table 6:** Effect of dietary APPs supplementation on lipid metabolism-related gene mRNA levels in the liver of finishing pigs.

Items	CON	0.04% APPs	0.08% APPs
*ACC*	1.00 ± 0.06	0.85 ± 0.11	1.03 ± 0.15
*FAS*	1.00 ± 0.02	1.00 ± 0.04	1.10 ± 0.03
*HSL*	1.00 ± 0.12 ^b^	1.67 ± 0.12 ^a^	1.37 ± 0.14 ^a^
*CPT1b*	1.00 ± 0.07 ^b^	1.57 ± 0.08 ^a^	1.75 ± 0.16 ^a^
*PPARα*	1.00 ± 0.06 ^b^	0.82 ± 0.06 ^b^	1.75 ± 0.17 ^a^
*HMG-CoAR*	1.00 ± 0.29	0.78 ± 0.08	0.81 ± 0.05
*CYP7A1*	1.00 ± 0.08 ^b^	1.39 ± 0.09 ^a^	1.64 ± 0.10 ^a^
*LDL-R*	1.00 ± 0.09 ^b^	1.16 ± 0.13 ^b^	2.05 ± 0.12 ^a^

^a, b^ within a row, different letters differ significantly at *P* < 0.05. APPs = Apple polyphenols; *ACC* = Acetyl-CoA carboxylase; *FAS* = Fatty acid synthase; *HSL* = Hormone sensitive lipase; *PPARα* = Peroxisome proliferator-activated receptor α; *CPT1b* = Carnitine palmitoyl transferase-1b; *HMG-CoAR* = 3-hydroxy-3-methylglutaryl coenzyme A reductase, *CYP7A1* = Cholesterol 7α-hydroxylase; *LDL-R* = Low-density lipoprotein receptor.

**Table 7:** Effect of dietary APPs supplementation on fatty acid composition in the liver of finishing pigs (%).

Items	CON	0.04% APPs	0.08% APPs
C14:0	0.25 ± 0.02	0.29 ± 0.06	0.19 ± 0.02
C16:0	17.40 ± 0.63 ^a^	15.03 ± 0.68 ^b^	15.10 ± 0.51 ^b^
C17:0	1.05 ± 0.22	1.21 ± 0.28	1.93 ± 0.45
C18:0	27.70 ± 0.77	29.92 ± 1.16	27.61 ± 0.53
C16:1	0.41 ± 0.02	0.35 ± 0.06	0.38 ± 0.04
C17:1	0.28 ± 0.01	0.25 ± 0.05	0.33 ± 0.07
C18:1n9	12.46 ± 0.47	14.58 ± 2.50	10.91 ± 0.38
C18:2n6	20.16 ± 0.81	20.18 ± 0.94	19.76 ± 0.41
C18:3n6	0.24 ± 0.01	0.21 ± 0.02	0.19 ± 0.02
C18:3n3	0.49 ± 0.04	0.52 ± 0.07	0.42 ± 0.04
C20:1n9	0.23 ± 0.01	0.22 ± 0.01	0.23 ± 0.01
C20:3n6	0.65 ± 0.04	0.64 ± 0.07	0.80 ± 0.06
C20:4n6	0.11 ± 0.01 ^a^	0.09 ± 0.01 ^b^	0.09 ± 0.01 ^b^
C20:5n3	0.71 ± 0.02	0.67 ± 0.05	0.67 ± 0.08
C22:6n3	0.94 ± 0.25	1.18 ± 0.21	1.11 ± 0.15
SFA ^1^	61.10 ± 0.36	61.80 ± 0.65	61.15 ± 0.29
MUFA ^2^	13.53 ± 0.48	13.27 ± 0.87	12.01 ± 0.46
PUFA ^3^	23.71 ± 0.75	23.26 ± 0.81	24.22 ± 0.57
Δ9-16 desaturase activity ^4^	0.02 ± 0.01	0.02 ± 0.01	0.02 ± 0.01
Δ9-18 desaturase activity ^5^	0.46 ± 0.02 ^a^	0.41 ± 0.03 ^ab^	0.39 ± 0.02 ^b^

^a, b^ within a row, different letters differ significantly at *P* < 0.05. APPs = Apple polyphenols. ^1^ SFA = C10:0 + C12:0 + C13:0 + C14:0 + C16:0 + C18:0 + C20:0 + C21:0 + C22:0 + C23:0. ^2^ MUFA = C14:1 + C15:1 +C16:1 + C17:1 + C18:1n9 + C20:1n9 + C22:1n9 +C24:1n9. ^3^ PUFA = C18:2n6 + C20:2 + C18:3n6 + C18:3n3 + C20:3n3 + C20:3n6 + C20:4n6 + C20:5n3 + C22:2 + C22:6n3 ^4^ Δ9-16 = C16:1 / C16:0. ^5^ Δ9-18= C18:1n9 / C18:0.
